# Effectiveness of Chatbot interventions for reducing caregiver burden: Protocol for a systematic review and meta-analysis

**DOI:** 10.1016/j.mex.2025.103272

**Published:** 2025-03-17

**Authors:** Ravi Shankar, Anjali Bundele, Amartya Mukhopadhyay

**Affiliations:** a Research and Innovation, Medical Affairs, Alexandra Hospital, Singapore, Singapore; b Division of Respiratory and Critical Care Medicine, Department of Medicine, National University Hospital, Singapore, Singapore

**Keywords:** Chatbots, Conversational agents, Artificial intelligence, Natural language processing, Caregiver burden, Systematic Review and Meta-analysis.

## Abstract

This protocol outlines a systematic review and meta-analysis examining the effectiveness of fully automated, AI-driven chatbot interventions in reducing subjective burden among informal caregivers. We will search 8 electronic databases (PubMed, Web of Science, Embase, CINAHL, MEDLINE, Cochrane Library, PsycINFO, Scopus) and grey literature sources from January 2010 to December 2024 for randomized controlled trials (RCTs) meeting predefined eligibility criteria. The primary outcome is caregiver burden, assessed using validated scales such as the Zarit Burden Interview. Secondary outcomes encompass caregiver mental health, quality of life, self-efficacy and care recipient status. Two reviewers will independently perform study selection, data extraction, risk of bias evaluation using Cochrane RoB 2 tool, and appraise certainty of evidence utilizing the GRADE approach. We will conduct random-effects meta-analyses, subgroup analyses, and meta-regression to compute pooled effect estimates and explore heterogeneity. If quantitative synthesis is precluded, narrative synthesis will be undertaken following SWiM guideline. Caregiver partners will provide input on interpretation and dissemination of findings.

• Protocol adheres to PRISMA-P reporting standards and will be prospectively registered in PROSPERO
• Graphviz code for replicating the systematic review methodology diagram is provided
• Review will yield critical evidence to guide development and implementation of chatbots into caregiver support services

## Graphical abstract

Graphical abstract depicting the key components of the systematic review protocol, including databases searched, eligibility criteria, screening process, data extraction, quality appraisal, data synthesis methods, and anticipated outputs

**Figure:**
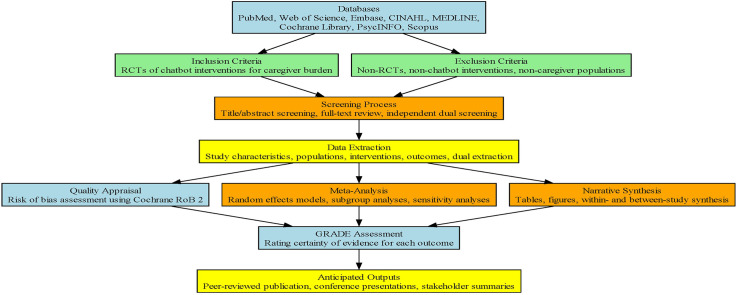

Specifications table

**Table:** 

Subject area:	Medicine and Dentistry
More specific subject area:	Digital health interventions for caregivers
Name of your method:	Systematic Review and Meta-analysis
Name of your protocol:	Effectiveness of Chatbot Interventions for Reducing Caregiver Burden: Protocol for a Systematic Review and Meta-Analysis
Reagents/tools:	Not applicable
Experimental design:	This systematic review will evaluate the effectiveness of fully automated, AI-driven chatbot interventions for reducing subjective burden among informal caregivers compared to inactive controls or non-chatbot active comparators. The primary outcome is caregiver burden, assessed using validated scales like the Zarit Burden Interview. Secondary outcomes include caregiver mental health, quality of life, self-efficacy, and care recipient status.
	We will systematically search 8 electronic databases (PubMed, Web of Science, Embase, CINAHL, MEDLINE, Cochrane Library, PsycINFO, Scopus) and grey literature sources from 2010 to December 2024. Study selection, data extraction, risk of bias assessment (Cochrane RoB 2 tool), and certainty of evidence appraisal (GRADE approach) will be conducted independently by two reviewers. Random-effects meta-analyses will be performed to calculate pooled effect estimates. Subgroup analyses and meta-regression will explore potential effect modifiers related to caregiver, care recipient, chatbot, and study characteristics. If quantitative synthesis is not feasible, narrative synthesis will be conducted following SWiM guidelines. Caregiver partners will provide input on interpretation and dissemination of findings.
Trial registration:	PROSPERO: CRD42024628606
Ethics:	Ethical approval is not required as this review analyzes previously published aggregate data. The results will be disseminated through a peer-reviewed publication.
Value of the Protocol:	- Establishes a rigorous, pre-specified methodology for evaluating the evidence on chatbot interventions for reducing caregiver burden, minimizing bias and enhancing transparency - Addresses a critical evidence gap in the rapidly evolving field of AI-powered caregiver support technologies - Informs evidence-based development and implementation of chatbot interventions into caregiver support services - Engages caregiver partners in interpreting findings to maximize relevance and impact - Provides Graphviz code for reproducing the systematic review methodology diagram, enabling replication by other researchers

## Background

Informal caregivers play a vital role in supporting individuals with health conditions and disabilities, but often face significant caregiver burden - the physical, emotional, social and financial strain of providing care. In the United States alone, over 50 million adults serve as informal caregivers, providing care worth over $500 billion annually [1]. Across OECD countries, around 13 % of adults are informal caregivers, with increasing prevalence expected due to population aging, rising disability rates, and shifts towards home and community-based care [2].

While caring for a loved one can be meaningful, it often entails significant costs to caregivers' well-being. Studies suggest 40–70 % of caregivers experience high levels of burden [3,4], with higher prevalence among those caring for people with dementia, mental illness, and complex needs [5,6]. Caregiver burden is associated with psychiatric morbidity, stress biomarkers, health risk behaviors, cardiovascular disease, premature mortality, and elder abuse [[7], [8], [9], [10]]. Burden also diminishes caregivers' ability to provide quality care, resulting in adverse care recipient outcomes such as malnutrition, hospitalizations, and unmet needs [11,12]. Lost productivity due to caregiving is estimated at $67 billion yearly in the US [13].

Despite their essential contributions, most caregivers lack adequate support. Unmet needs are pervasive for respite, information, skills training, and coping resources [14,15]. Numerous barriers impede access to support, including fragmented services, financial strain, time constraints, transportation challenges, and limited awareness [16]. The COVID-19 pandemic has exacerbated caregiving difficulties while disrupting usual supports [17].

Digital technologies, particularly chatbots, present a promising avenue to fill gaps in caregiver support. Chatbots are artificial intelligence (AI) software programs that engage in human-like dialogue, simulating natural conversations by understanding queries and providing relevant responses [18]. Leveraging natural language processing, machine learning, and other AI techniques, chatbots can offer tailored information, skills training, self-care tools, and emotional support - all personalized to each caregiver's unique situation, preferences, and communication style [19].

A growing body of evidence supports the acceptability and effectiveness of chatbots for mental health [[20], [21], [22]], health promotion [23], and chronic disease management [24]. Randomized trials have shown chatbots to reduce depression, anxiety, and stress [[25], [26], [27]]. Systematic reviews highlight benefits in increasing access to care, engaging hard-to-reach groups, and empowering self-management [[28], [29], [30]].

Key advantages of chatbots for caregiver support include:

• On-demand 24/7 access via web and mobile apps, overcoming logistical barriers
• Anonymity and self-paced interactions to reduce stigma and encourage disclosure
• Personalized psychoeducation, coping techniques, and motivation tailored to individual needs
• Capacity to learn communication styles and establish empathetic therapeutic alliance
• Cost-effectiveness and scalability to provide population-level support

However, research on chatbots specifically for caregiver burden is nascent. Prior reviews have examined chatbots for health education [31], mental health [20,21], and behavior change [32], but none have comprehensively synthesized evidence on reducing caregiver burden. The only pertinent review focused on dementia caregivers and included one chatbot study within a broader analysis of technologies [33].

## Description of protocol

To advance the field, an up-to-date review is needed to rigorously evaluate the effectiveness of chatbots for alleviating burden across diverse caregiver populations. By appraising study quality, exploring moderators and mechanisms of effect, and characterizing research gaps, such a review could accelerate the development and implementation of high-quality, impactful chatbots for caregiver support. Findings could also inform policies and investment priorities to promote equitable chatbot access and mitigate potential disparities.

### Objectives

This systematic review aims to assess the effectiveness of chatbot interventions in reducing subjective burden among informal caregivers. The objectives are:

1. To estimate the effects of chatbot interventions on caregiver burden and secondary outcomes (mental health, quality of life, self-efficacy, and care recipient status), compared to inactive controls or active non-chatbot comparators.
2. To explore potential effect modifiers, including:

○ Caregiver factors: demographics, caregiving relationship, duration and intensity
○ Care recipient factors: health condition, symptom severity, care needs
○ Chatbot characteristics: embodiment, communication mode, AI architecture, scope
○ Comparator type: inactive (waitlist, usual care) vs. active (education, support group)
○ Study design: delivery mode, duration of follow-up

3. To summarize evidence on the implementation of chatbot interventions for caregivers, encompassing user engagement, acceptability, usability, and economic evaluations.
4. To assess the risk of bias of individual studies and overall certainty of evidence for each outcome using established tools.
5. To identify key research gaps, methodological limitations, and recommendations for optimizing future chatbot interventions for caregivers.

## Description of protocol

### Methods

This systematic review will be conducted in accordance with the Preferred Reporting Items for Systematic Reviews and Meta-Analyses (PRISMA) 2020 statement [34]. The protocol adheres to the PRISMA-P checklist [35] and will be registered in the International Prospective Register of Systematic Reviews (PROSPERO).

### Eligibility criteria

We will select studies based on the following PICO (Population, Intervention, Comparison, Outcome) criteria:

*Population*: Informal adult caregivers (aged ≥18 years) of individuals with health conditions, disabilities, or functional limitations. Caregivers may support care recipients of any age, from children to older adults. Studies with ≥80 % informal caregivers will be included, unless outcome data are reported separately for informal caregivers.
*Intervention*: AI chatbot interventions that caregivers primarily interact with via natural language dialogue (text or speech). We will include fully automated, standalone chatbots, but exclude human-operated or multi-component chatbots where the chatbot is not the main intervention. There will be no restrictions on technical features, delivery mode, or intended use of the chatbots.
*Comparison*: Inactive controls (e.g., waitlist, usual care, attention control) or active non-chatbot interventions (e.g., static online education, support group, therapist consultation). Studies with factorial designs that preclude isolating chatbot effects will be excluded.
*Outcomes*: The primary outcome is caregiver burden, assessed using validated instruments such as the Zarit Burden Interview [36], Caregiver Strain Index [37], or Caregiver Burden Inventory [38]. If a study uses multiple scales, the Zarit Burden Interview will be prioritized as it is the most commonly used measure. Secondary outcomes are:

• Caregiver mental health: depression, anxiety, stress, distress
• Quality of life and positive aspects of caregiving
• Self-efficacy for managing care challenges
• Care recipient clinical, behavioral, and social outcomes
• Adverse events or unintended negative consequences

We will consider both post-intervention and follow-up (≥1 month post-intervention) time points.

Implementation outcomes will also be extracted to inform real-world applicability, including:

• User engagement metrics (e.g., uptake, usage frequency and duration, retention)
• Acceptability, satisfaction, perceived benefits and challenges
• Technical performance (e.g., responsiveness, errors, downtime)
• Economic evaluations (e.g., costs, cost-effectiveness, return on investment)

*Study Design*: We will include randomized controlled trials (RCTs), encompassing parallel group, cluster randomized, and crossover trials. For crossover RCTs, only the first treatment period will be included to avoid carryover effects. Non-randomized studies will be excluded as our aim is to evaluate causal effectiveness.
*Setting and Language:* We will include studies conducted in any country and setting. While the primary focus will be on English language publications, we will also consider non-English articles that can be reliably translated using professional translation services or Google Translate to minimize language bias.
*Year of Publication*: To capture recent, AI-driven chatbots, we will include studies published from 2010 onwards.

### Information sources and search strategy

We will systematically search eight electronic databases from their inception to December 2024. These databases include PubMed, Web of Science, Embase, CINAHL, MEDLINE, The Cochrane Library, PsycINFO, and Scopus.

The search strategy will be developed in consultation with a medical librarian and use a combination of keywords and controlled vocabulary terms (e.g., MeSH) related to chatbots, conversational agents, artificial intelligence, caregivers, and burden. A preliminary search string for PubMed is:

(chatbot* OR ``conversational agent*'' OR ``conversational AI'' OR ``virtual agent*'' OR ``dialog* system*'') AND (caregiv* OR care-giv* OR ``care provider*'' OR ``informal caregiver*'' OR ``family caregiver*'' OR ``parental caregiver*'') AND (burden OR strain OR stress OR distress OR ``quality of life'')

Search strategies will be optimized for each database. To capture grey literature, we will search Google Scholar, ProQuest Dissertations & Theses, and ClinicalTrials.gov. Reference lists of pertinent reviews and included studies will be hand-searched.

### Study selection

**Fig. 1:**
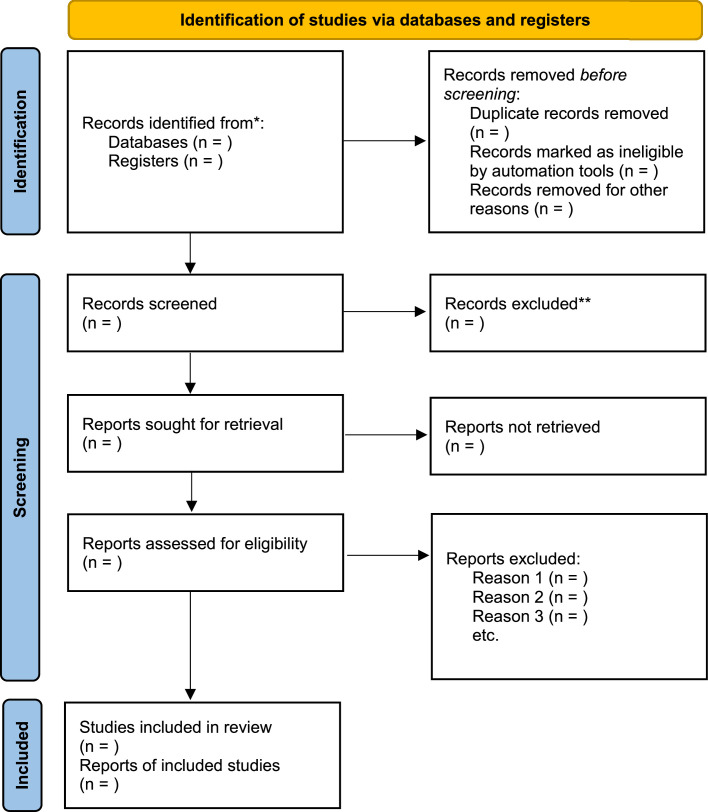
PRISMA flow diagram.

Two reviewers will independently screen titles and abstracts of all records in Covidence systematic review software [39]. Full texts of potentially eligible studies will be retrieved and reviewed independently against inclusion criteria. Reasons for exclusion will be recorded. Disagreements will be resolved through discussion or adjudication by a third reviewer. The study selection process will be illustrated in a PRISMA flow diagram (Fig. 1).

### Data extraction

Two reviewers will independently use a standardized form to extract data from the included studies. Any discrepancies will be resolved by consensus or, if needed, by involving a third reviewer. The data extraction form will be piloted on a sample of 3–5 included studies to ensure consistency and comprehensiveness before full data extraction begins. For missing or ambiguous information, corresponding authors will be contacted for clarification. The primary variables will encompass key study characteristics (e.g., design, setting, sample size), caregiver and care recipient demographics (e.g., age, gender, primary diagnosis), chatbot intervention details (e.g., theoretical underpinning, AI architecture, communication style), comparator intervention parameters, outcome measures, and relevant implementation factors. We will also note each study's main conclusions and any potential conflicts of interest. (Details in Appendix A.)

### Risk of bias assessment

Risk of bias will be assessed independently by two reviewers using the revised Cochrane Risk of Bias tool for randomized trials (RoB 2) [40]. This tool evaluates bias arising from the randomization process, deviations from intended interventions, missing outcome data, outcome measurement, and selective reporting. Each domain is judged as low risk, some concerns, or high risk. These inform an overall risk of bias judgement for each outcome as low, some concerns, or high risk. Discrepancies between reviewers will be resolved by consensus or a third reviewer. Risk of bias will be summarized graphically and inform interpretation of findings.

### Synthesis of results

We will provide a narrative synthesis of all included studies, summarizing their characteristics, findings, and limitations in text and tables. We will also seek to quantitatively synthesize results using random-effects meta-analyses where appropriate.

For the primary outcome of caregiver burden, we will calculate standardized mean differences (Hedges' g) and 95 % confidence intervals to compare chatbot and control groups. If multiple burden scales are reported, we will prioritize the measure specified as primary by study authors or the most prevalent scale across included studies. We will meta-analyze post-intervention and follow-up endpoints separately. For multi-arm trials, we will combine all relevant control arms to enable a single pairwise comparison. Dichotomous secondary outcomes will be analyzed as risk ratios.

Meta-analyses will be performed in RevMan software. The I² statistic will be used to assess the percentage of variability in effect estimates that is due to heterogeneity rather than sampling error, with values of 25 %, 50 % and 75 % suggesting low, moderate, and high levels respectively [41]. Sources of heterogeneity will be explored using subgroup analyses based on:

• Caregiver age (<65 vs. ≥65 years), gender, and race/ethnicity
• Caregiver-care recipient relationship (spouse vs. non-spouse)
• Care recipient condition (dementia vs. non-dementia) and severity
• Chatbot characteristics (embodiment, communication mode, AI architecture, scope)
• Comparator type (inactive vs. active)
• Study quality (low vs. high risk of bias)
• Geographic region (North America, Europe, Asia, Other)
• Publication date (before vs. in/after 2019)

If ≥10 studies are available, we will conduct random-effects meta-regression to identify predictors of intervention effects. For meta-regression analyses, we will require a minimum of 10 studies per covariate to ensure reliable estimates. Sensitivity analyses will exclude studies at high risk of bias for the outcome being analyzed, studies with potential outlier results, and studies with small sample sizes to assess the robustness of the findings [42].

If statistical pooling is not viable due to inconsistent reporting, high heterogeneity, or insufficient studies, we will use vote counting based on direction of effect and synthesize findings narratively [43]. We will summarize intervention characteristics, effects, and limitations in tables, structuring the synthesis around the pre-specified subgroup factors. Both within-study (i.e., between-arm) and between-study comparisons will be used to identify patterns in the results.

### Certainty of evidence

We will evaluate the certainty of evidence for each outcome using the Grading of Recommendations Assessment, Development and Evaluation (GRADE) approach [44]. GRADE assesses the confidence that the true effect lies close to the estimate, based on consideration of risk of bias, imprecision, inconsistency, indirectness, and publication bias. Outcomes are rated as high, moderate, low, or very low certainty. Two reviewers will independently judge each domain, with discrepancies resolved through discussion. Ratings and rationales will be presented in a summary of findings table.

### Ethics and dissemination

Ethical approval is not required for this systematic review as it only involves analysis of previously published, de-identified data. The review will be disseminated through publication in a peer-reviewed journal, conference presentations, and summaries tailored to caregiver organizations, chatbot developers, clinicians, policymakers, and funding agencies. The protocol will be registered in PROSPERO and uploaded to an open access repository. Any protocol amendments will be documented with rationale and date.

### Patient and public involvement

Caregiver partners will not be directly involved in conducting the systematic review, but we will seek their input on interpreting and disseminating the findings to ensure relevance and reach. We will invite 2–3 caregivers to review a lay summary of the results and provide feedback on key messages, preferred communication channels, and suggestions for increasing the accessibility and impact of the review. Their contributions will be acknowledged in the final manuscript.

### Protocol validation

This systematic review will provide the most comprehensive and rigorous evidence to date on the effectiveness of chatbot interventions for reducing informal caregiver burden. By synthesizing data from randomized trials, critically appraising study quality, exploring effect modifiers, and characterizing research gaps, the review will yield actionable insights to guide the development, evaluation, and implementation of chatbots for caregiver support.

The review will make several novel contributions to research and practice. First, as the only review focused specifically on chatbots for caregiver burden, it will establish the state-of-the-art in this emerging field. Second, the inclusion of secondary and implementation outcomes will shed light on broader impacts and real-world feasibility, complementing efficacy data. Third, the exploration of moderators will identify which types of chatbots work best for whom and under what conditions, informing precision approaches. Fourth, the application of GRADE will provide policymakers and practitioners with a transparent, systematic assessment of evidence certainty to guide decision-making. Finally, the identification of research gaps and methodological limitations will set an agenda for future studies.

Strengths of the review methods include pre-registration of a publicly available protocol, an exhaustive search strategy, independent study selection and data extraction in duplicate, use of validated tools for quality appraisal, application of gold-standard GRADE methodology, and adherence to PRISMA reporting guidelines. These features will minimize bias and enhance transparency and reproducibility.

The review findings will yield several important implications. From a clinical perspective, establishing the effectiveness of specific chatbot features and approaches for alleviating caregiver burden could facilitate their integration into caregiver support programs and clinical guidelines. Economic data could inform coverage and reimbursement policies. Policymakers and health system leaders could use the findings to guide investments in chatbot infrastructure, training, and evaluation. For researchers and technology developers, understanding current best practices and limitations could incentivize user-centered co-design of chatbots that optimize efficacy, engagement, and equity. Across stakeholder groups, accessible evidence summaries could raise awareness of chatbots as an emerging service delivery model.

In the context of population aging, rising caregiver demands, and ubiquitous technology access, chatbots represent a promising strategy to empower caregivers at scale. As highly accessible, affordable, and adaptive interventions, chatbots could help bridge pervasive gaps in caregiver support while personalizing care to the unique needs, preferences, and contexts of diverse caregivers. If found effective, chatbots could yield cascading benefits for care recipients, health systems, and societies by reducing caregiver burnout, improving care quality, preventing premature institutionalization, and promoting equitable health.

However, the ethical and social implications of chatbots for caregiving warrant proactive consideration. Chatbots may perpetuate inequities if their development and deployment does not prioritize inclusivity, cultural competence, and digital literacy. Over-reliance on chatbots could delay recognition of complex caregiver needs and timely referral to human providers. Establishing guidelines for transparency, safety, and accountability in chatbot use will be critical.

Ultimately, this systematic review will provide researchers, practitioners, and decision-makers with a comprehensive, critical synthesis of current evidence on chatbots for caregiver support. By identifying effective intervention components and implementation strategies as well as key research gaps and priorities, the review will advance the field towards developing and scaling high-quality, equitable, and caregiver-centered chatbot services. Such evidence-based innovations in caregiver support have the potential to transform the lives of the growing population of informal caregivers worldwide - a public health imperative in the coming decades.

## Limitations

Some limitations warrant consideration. Despite our inclusion of non-English language articles when translation is feasible, language barriers may still limit full representation of studies from non-Anglophone contexts. The focus on randomized trials, while necessary to support causal claims, may come at the cost of excluding relevant real-world evidence from non-randomized studies. The 2010 publication cut-off will improve comparability of chatbot technologies but may omit earlier, relevant studies. Heterogeneity in populations, interventions, comparators, and outcomes may hinder meta-analysis and introduce uncertainty in subgroup and sensitivity analyses. Narrative synthesis methods will be used if statistical pooling is not appropriate.

## CRediT author statement

**Ravi Shankar:** Conceptualization, Methodology, Writing - Original Draft, Visualization. **Anjali Bundele:** Methodology, Writing - Review & Editing. **Amartya Mukhopadhyay:** Methodology, Writing - Review & Editing.

## Declaration of competing interest

The authors declare that they have no known competing financial interests or personal relationships that could have appeared to influence the work reported in this paper.

## Data Availability

No data was used for the research described in the article.

## Appendix Supplementary materials

Image, application 1
