# Supplementary material for: Effectiveness of Chatbot interventions for reducing caregiver burden: Protocol for a systematic review and meta-analysis
Source: MethodsX. 2025 Mar 17;14:103272. doi: 10.1016/j.mex.2025.103272 (PMC11978356; doi:10.1016/j.mex.2025.103272)
Supplement: Supplementary file 1 [file mmc1.docx]

**Appendix A. Data extraction form**

| **S. No.** | **Categories** | **Variables** |
| --- | --- | --- |
| 1 | **Study Characteristics** | - **First author** - **Publication year** - **Country** - **Setting** (community, clinic, online) - **Study design** - **Sample size** (total and per group) - **Study duration** - **Funding source** |
| 2 | **Population** | **Caregiver Characteristics** - Mean age (SD) - Gender (n, % female) - Race/ethnicity (n, % per category) - Education level (n, % per category) - Employment status (n, % employed) - Relationship to care recipient (n, % spouse, child, parent, other) - Cohabitation with care recipient (n, % yes) - Mean caregiving duration in months (SD) - Mean caregiving hours per week (SD) - Baseline burden score, mean (SD)  **Care Recipient Characteristics** - Mean age (SD) - Gender (n, % female) - Primary diagnosis (n, % per condition) - Disease stage/severity (n, % per category if available) - Symptom severity score, mean (SD) - Level of functional dependence (n, % per category if available) - Presence of behavioral symptoms (n, % yes) |
| 3 | **Interventions** | **Chatbot Intervention** - Name of chatbot - Developer of chatbot - Theoretical basis - Chatbot architecture (retrieval-based, generative, rule-based, hybrid) - Underlying algorithm (e.g., natural language processing, deep learning) - Knowledge base (e.g., expert rules, training data) - Input modality (text, speech, multimodal) - Output modality (text, speech, nonverbal cues) - Embodiment (avatar, disembodied) - Dialogue management (finite state, frame-based, agent-based) - Scope of support (psychoeducation, skills training, coping strategies, etc.) - Access (smartphone app, web-based, messaging platform) - Recommended usage frequency and duration - Actual usage metrics, mean (SD) if reported - Personalization (static, dynamic, user-initiated, proactive) - Relational behaviors (empathy, self-disclosure, humor, meta-relational) - Gamification and engagement features - Security and privacy features  **Comparator Intervention(s)** - Type (waitlist control, usual care, attention control, active non-chatbot) - Delivery format (e.g., in-person, online, print materials) - Content and features - Providers (e.g., nurse, social worker, trained lay coach) - Dose (frequency and duration) |
| 4 | **Outcomes** | **Caregiver Burden** - Instrument used - Score range and direction - Reliability and validity - Timing of assessment - Scores, mean (SD) (Baseline, Post-intervention, Follow-up) - Effect size (post-intervention between-group difference) - *p*-value  **Caregiver Mental Health** - Instrument used (depression, anxiety, stress) - Scores, mean (SD) - Effect size - *p*-value  **Caregiver Quality of Life** - Instrument used - Scores, mean (SD) - Effect size - *p*-value  **Caregiver Self-Efficacy** - Instrument used - Scores, mean (SD) - Effect size - *p*-value  **Care Recipient Outcomes** - Instruments used (behavioral symptoms, health status, quality of life) - Scores, mean (SD) - Effect sizes - *p*-values  **Adverse Events/Harms** - Description of event - Number (%) per group - Severity - Relationship to study |
| 5 | **Implementation Outcomes** | **Engagement** - Uptake/enrollment rate - Actual usage frequency, mean (SD) - Actual usage duration per session, mean (SD) - Total intervention exposure/dose received - Retention rate (% completing intervention) - Reasons for non-use or dropout  **User Experience** - Acceptability (satisfaction ratings, qualitative feedback) - Usability (ease of use ratings, errors, help requests) - Perceived benefits - Perceived barriers/challenges  **Technical Performance** - System responsiveness (e.g., latency, up-time) - Error rate - Accuracy of speech recognition and natural language understanding  **Cost** - Estimated cost per user - Cost-effectiveness (incremental cost-effectiveness ratios) - Projected return on investment |
| 6 | **Conclusions and Limitations** | - Key findings - Authors’ conclusions - Limitations - Conflicts of interest - Funding source |
